# Comprehensive Analysis of the Transcriptome-wide m6A Methylome in Lung Adenocarcinoma by MeRIP Sequencing

**DOI:** 10.3389/fonc.2022.791332

**Published:** 2022-07-11

**Authors:** Wenli Mao, Qingzhen Yu, Kefeng Wang, Qiang Ma, Yuxin Zheng, Guojun Zhang, Wei Luo, Nianwu Wang, Yukun Wang

**Affiliations:** ^1^ Department of Pharmacology, School of Medicine, Southern University of Science and Technology, Shenzhen, China; ^2^ Medical Research Center, Southern University of Science and Technology Hospital, Shenzhen, China; ^3^ Nutrition Department, Southern University of Science and Technology Hospital, Shenzhen, China; ^4^ Department of Clinical Laboratory, Southern University of Science and Technology Hospital, Shenzhen, China; ^5^ Department of Pharmacy, Southern University of Science and Technology Hospital, Shenzhen, China

**Keywords:** lung adenocarcinoma, m6A, MeRIP-seq, GPRIN1, prognosis

## Abstract

N6-methyladenosine (m6A) is the most abundant internal modification on eukaryotic mRNAs. There is increasing evidence that m6A plays a key role in tumor progression, so it is important to analyze m6A modifications within the transcriptome-wide in lung adenocarcinoma (LUAD). Three pairs of LUAD samples and tumor-adjacent normal tissues were obtained from the South University of Science and Technology Hospital. And then methylated RNA immunoprecipitation sequencing (MeRIP-seq) and RNA sequencing (RNA-seq) were used to identify differential m6A modifications between tumor and tumor-adjacent normal tissues. We identified 4041 aberrant m6A peaks, of which 1192 m6A peaks were upregulated and 2849 m6A peaks downregulated. It was found that genes with the dysregulated m6A peaks were enriched in the pathways in cancer, Rap1 signaling pathway, and insulin resistance. Additionally, 612 genes with abnormal regulation of m6A peaks and RNA expression were identified by combining MeRIP-seq and RNA-seq data. Through KEGG analysis, the 612 genes were enriched in cancer-related signaling pathways, such as the cGMP-PKG signaling pathway, and the Rap1 signaling pathway. What’s more, GSEA enrichment analysis showed these genes were enriched in cell cycle phase transition, cell division, cellular response to DNA damage stimulus, and chromosome organization. To further explore the relationship between differential m6A modified genes and clinical parameters of LUAD patients, we searched The Cancer Genome Atlas (TCGA) and identified 2 genes (FCRL5 and GPRIN1) that were associated with the prognosis and diagnosis of LUAD patients. Furthermore, we found a positive correlation between GPRIN1 and m6A reader YTHDF1 in the GEPIA2 database. It was verified that YTHDF1 binds to GPRIN1 mRNA and regulates its expression. Our study results suggest that m6A modification plays important role in the progression and prognosis of LUAD and maybe a potential new therapeutic target for LUAD patients in the future.

## Introduction

RNA methylation mostly regulates gene expression at the posttranscriptional level, which is considered to be another layer of epigenetic regulation similar to DNA methylation and histone modification. Recently, a variety of molecular modifications of RNA (including rRNA, tRNA, snRNA, mRNA, and long non-coding RNA) have been discovered (1). The ways of RNA methylation modification include N6-methyladenosine (m6A), 5-methylcytosine (m5C), N1-methyladenosine (m1A), 5-hydroxymethylcytosine (5hmC), N6, 2′-Odimethyladenosine (m6Am), 7-methylguanine (m7G) (2), of which m6A modification is the most abundant type in eukaryotic (3). The m6A modification is a methylation modification on the RNA level that was first discovered in the 1970s (4). The m6A high-throughput sequencing study revealed that m6A is a selective modification based on the recruitment of specific mRNAs. The m6A modification mainly takes place on the RRACH (R = G or A, H = A, C or U) consensus sequence (5–7), of which G(m6A)C (70%) or A(m6A)C (30%) (8), and is enriched near the 3’UTR, and stop codon (9). m6A modification is a dynamic and reversible methylation modification regulated by the m6A regulators, which is mainly composed of “writers” (METTL3, METTL14), and “erasers” (ALKBH5, FTO), and “readers” (YTHDFs, YTHDCs, IGF2BPs) (10). Increasing studies have confirmed that m6A modification plays an important role in malignant tumors (11, 12). For instance, m6A reader YTHDF1 promotes the proliferation, migration, invasion, and cell cycle of hepatocellular carcinoma cells by activating the PI3K/AKT/mTOR signaling pathway (13). Additionally, METTL3 mediates m6A modification of circCUX1 and stabilizes its expression, which binds caspase1 to reduce the release of inflammatory factors, leading to radiotherapy tolerance in hypopharyngeal squamous cell carcinoma (HPSCC) (14).

Lung cancer is the most common malignant tumor, with the highest mortality rate in the world, and only 21% of the 5-year survival rate (15). LUAD is the most common histological type of lung cancer, accounting for about 40% (16). In recent years, some studies have reported that m6A modification plays an important role in the progression of lung adenocarcinoma (17). Li et al. (16) revealed that YTHDF2 was involved in the development of LUAD *via* promoting the attenuation of AXIN1 mRNA to activate the Wnt/β-catenin signaling pathway, which may be a potential therapeutic target for LUAD. In addition, miR-600 induces degradation of METTL3 mRNA by targeting the 3’UTR of METTL3 mRNA and leads to inactivation of the PI3K/AKT pathway related to cell growth and survival (18). What’s more, METTL3 promotes the translation of YAP mRNA and increases YAP activity by regulating the MALAT1-miR-1914-3p-YAP axis, inducing drug resistance and metastasis of A549 cells (19). Many studies have described the important role of m6A modification in LUAD. However, the transcriptome-wide m6A methylome of LUAD has not been determined.

In this study, we identified differential methylated peaks and expressed genes through MeRIP-seq and RNA-seq. Furthermore, 2 genes with significant changes in both the m6A modification and expression associated with prognosis and diagnosis of LUAD were discovered by additional TCGA clinical data analysis.

## Materials and Methods

### Patients and Samples

Three pairs of LUAD samples and tumor-adjacent normal tissues were obtained from the South University of Science and Technology Hospital. The LUAD samples and tumor-adjacent normal tissues were immediately collected and separated into centrifuge tubes after surgery. These samples were examined by experienced pathologists who confirmed the diagnosis of disease samples. All tissues were stored at −80°C until RNA isolation. This study was approved by the Ethics Committee of the South University of Science and Technology Hospital.

### MeRIP Sequencing and RNA Sequencing

Total RNA was isolated and purified using TRIzol™ Reagent (Invitrogen™, cat. no15596018) following the instructions of the manufacturer. Total RNA was chemically broken into 200nt fragments. The product was added to anti-N6-Methyladenosine (m6A) antibody (Sigma-Aldrich, cat. no ABE572), protein A-magnetic beads (Invitrogen™, cat. no 10002D), protein G-magnetic beads (Invitrogen™, cat. no 10004D), which was then mixed and incubated overnight. RNA was extracted by phenol-chloroform lysate to afford the purified product. A portion of the initial fragmented RNA was used as the input RNA-seq library for MeRIP-seq. Ribosomal RNA was removed from the products. The first-strand cDNA was synthesized by the SMART principle, and enriched library fragments were amplified by PCR. Ultrafine RNA methylation m6A detection library fragments were obtained by DNA purification of magnetic bead library fragments. Bioptic Qsep100 Analyzer was used to perform a quality inspection on the library and detect whether the size distribution of the library is consistent with the theoretical size. NovaSeq high-throughput sequencing platform and PE150 sequencing mode were used.

### Cell Culture and Transfection

Lung adenocarcinoma cell line A549 cells were cultivated in 1640 medium containing 10% fetal bovine serum (FBS) (ExCell Bio Inc., Shanghai, China) and 100 U/mL penicillin-streptomycin mixture (Gibco, Grand Island, NY, USA). The cells were maintained at 37°C in a humidified atmosphere of 95% air and 5% CO2. YTHDF1 siRNA was designed and constructed by Genechem Bio (Shanghai, China). In transfection, siRNAs were transfected by LipoRNAiMAX Transfection Reagent (Invitrogen) according to product instructions. Briefly, siRNAs were diluted in the 100 μL Opti-MEM (Gibco) at 10 nmol/L and mixed with 2.5 μL Lipofectamine RNAi Max. After incubation at room temperature for 18 min, the mixture was added to the cell culture medium, and the cells were collected after 48 h. The sequences of the targets were as follows: YTHDF1-Homo-370 sense 5’- GGAUACAGUUCAUGACAAUTT-3’, antisense 5’-AUUGUCAUGAACUGUAUCCTT-3’; YTHDF1-Homo-1076 sense 5’-CCUCCACCCAUAAAGCAUATT-3’, antisense UAUGCUUUAUGGGUGGAGGTT-3’; YTHDF1-Homo-1503 sense 5’-GCUCCAUUAAGUACUCCAUTT-3’, antisense 5’-AUGGAGUACUUAAUGGAGCTT-3’.

### RNA Isolation and RT-qPCR

Total RNA was extracted from A549 cells using Trizol (Takara, Japan) according to the manufacturer’s instructions. For RT-qPCR, RNA was reverse transcribed to cDNA by using a reverse transcription kit (Takara, Japan). The levels of RNA transcripts were analyzed by the Jena qTOWER3 RT-qPCR system (Jena, Germany). The sequences of the primers were as follows: GAPDH forward primer, 5’-CTCCTCCTGTTCGACAGTCAGC-3’, reverse primer 5’-CCCAATACGACCAAATCCGTT-3’; YTHDF1 forward primer, 5’-GCACACAACCTCCATCTTCG-3’, reverse primer 5’-AACTGGTTCGCCCTCATTGT-3’; GPRIN1 forward primer 5’-AAAGCAGGCCGATTCCACTTC-3’, reverse primer 5’-TCCTTCCTCGGTGACACTGTA-3’.

### RIP-qPCR

The collected cell precipitates were coprecipitated by EZ-Magna RIP™ RNA-Binding Protein Immunoprecipitation Kit (Millipore 17-701) following the manufacturer’s procedure. Cells were lysed in a moderate volume RIP lysis buffer on ice for 5 min, and then the lysates were moved to -80°C overnight. Lysates were centrifuged at 4°C, and 12,000 rpm for 30 min to obtain the supernatant. 5μg YTHDF1 antibody or rabbit IgG was conjugated to protein A/G magnetic beads on a shaker for 30 min at room temperature. Magnetic beads containing antibodies were added to the supernatant, followed by rotating overnight at 4°C. TRIzol LS reagent (Invitrogen) was used to extract the RNA according to the manufacturer’s instructions. RT-qPCR follows the above method.

### Public Databases and Analysis

The Database for Annotation, Visualization and Integrated Discovery (DAVID) database (https://david.ncifc rf.gov/) was used in Gene Oncology (GO) functional enrichment analysis and Kyoto Encyclopedia of Genes and Genomes (KEGG) pathway analysis. 594 LUAD patients’ RNA-seq data were downloaded from The Cancer Genome Atlas (TCGA) database (https://portal.gdc.cancer.gov/). The expression levels of genes were analyzed using the R package DESeq2 [version 1.26.0]. Then, 526 LUAD patients including primary tumor, lymph node metastasis, distant metastasis, and clinical stage were selected for the further study. A univariate Cox regression model was adopted to calculate the hazard ratios (HRs) of the genes’ overall survival. Kaplan-Meier analysis was used to construct survival curves, and the Cox regression model was utilized to estimate the significance of the differences. ROC curves were constructed to estimate the accuracy of genes in predicting outcomes. YTHDF1-RIP-seq data was downloaded from the NCBI Gene Expression Omnibus (GEO) database under the accession number GSE136433.

## Results

### Overview of the m6A Methylation Map in LUAD

**Figure 1:**
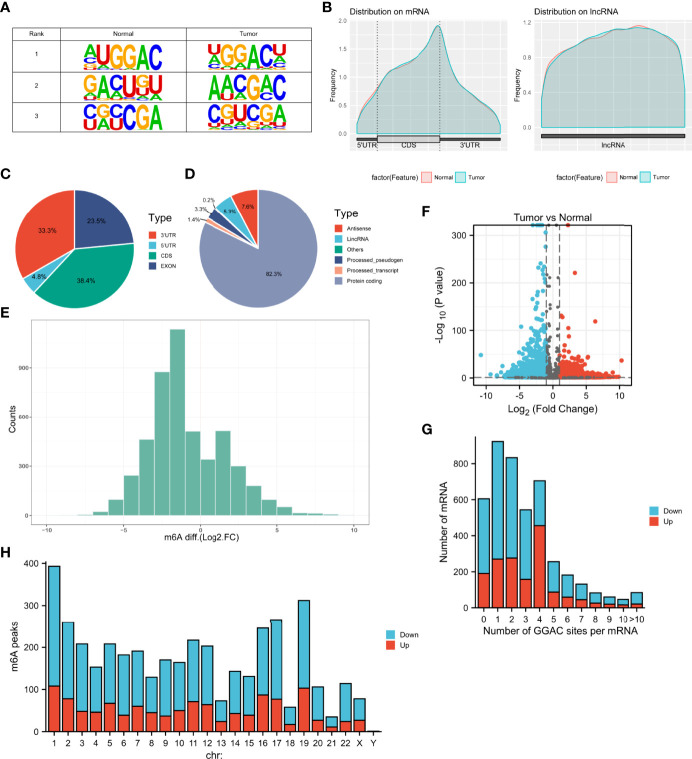
Characteristics of m6A methylation in LUAD. **(A)** The sequence logo showed the top three m6A motifs. **(B)** The distribution of m6A peaks in normal and tumor tissues. **(C, D)** Pie charts showing the distribution of differently methylated m6A peaks. **(E)** Histogram showing the changes in m6A enrichment between normal and tumor samples. **(F)** Volcano plots show significantly different m6A peaks. **(G)** The distribution of altered GGAC sequence per mRNA. **(H)** The distribution of differentially methylated m6A peaks in human chromosomes. Fold change ≥ 2 and *P* < 0.05.

MeRIP-seq analysis of tumor tissue and tumor-adjacent normal tissue from three LUAD patients was performed. The sequence logo showed the top three m6A motifs enriched from altered m6A peaks and the “GGAC” sequence was ranked in the first place **(** **Figure 1A**). The distribution of m6A peaks in normal and tumor tissues was further investigated. **Figures 1B–D** shows that the distribution of m6A signals around mRNA and lncRNA were comparable in the two classes of tissue samples. Also, m6A peaks in mRNA both in tumor and normal tissues were mainly enriched in the coding sequence near the stop codon. It was found that their m6A mRNA methylation was reduced globally in the tumor compartment compared with adjacent, normal control tissues (**Figure 1E**). In **Figure 1F**, by comparing with normal tissues, tumor tissues had 1192 significantly upregulated m6A peaks, corresponding to transcripts of 1092 genes, as well as 2849 significantly downregulated m6A peaks, representing transcripts of 2415 genes. The top 20 altered m6A peaks are listed in **Table 1**. By analyzing the distribution of m6A peaks per mRNA, one m6A peak in the majority of mRNAs was found (**Figure 1G**). All altered m6A peaks were mapped to human chromosomes. Dysregulated m6A peaks were also observed in all chromosomes, particularly in chr1, chr2, chr16, chr17, and chr19 (**Figure 1H**).

**Table 1:** Top 20 differently methylated m6A peaks in Tumor vs Normal.

Gene ID	Gene name	Chromosome	Peak start	Peak end	Log2 FC	Peak region	P value
ENSG00000164107	HAND2	chr4	173528770	173528979	-10.8	CDS	6.30957E-49
ENSG00000259721	AC090877.2	chr15	32718078	32718318	10.3	exon	1.58489E-37
ENSG00000214353	VAC14-AS1	chr16	70772801	70773131	9.85	exon	0.004365158
ENSG00000188385	JAKMIP3	chr10	132182472	132182922	9.5	3'UTR	0.005495409
ENSG00000128694	OSGEPL1	chr2	189755305	189755545	-9.34	CDS	1.62181E-08
ENSG00000231574	LINC02015	chr3	177898896	177899224	9.08	exon	0.005495409
ENSG00000164778	EN2	chr7	155458128	155458456	8.97	5'UTR	1.99526E-12
ENSG00000136002	ARHGEF4	chr2	130916358	130916809	8.67	CDS	0.009772372
ENSG00000137571	SLC05A1	chr8	69672400	69673269	8.62	3'UTR	0.007585776
ENSG00000169258	GPRIN1	chr5	176596219	176596548	8.43	3'UTR	5.62341E-05
ENSG00000147119	CHST7	chrX	46598169	46598408	-8.39	3'UTR	0.000616595
ENSG00000155657	TTN	chr2	178535385	178535626	-8.39	CDS	1.94984E-06
ENSG00000204776	IGKV1OR-3	chr9	65771144	65771434	8.39	exon	0.008912509
ENSG00000122728	TAF1L	chr9	32635100	32635400	8.38	exon	0.012022644
ENSG00000127530	OR7C1	chr19	14799720	14800141	8.14	exon	0.010471285
ENSG00000215146	BX322639.1	chr10	42336527	42336527	8.04	exon	2.39883E-05
ENSG00000179532	DNHD1	chr11	6546403	6546614	7.97	CDS	0.001096478
ENSG00000232803	SLCO4A1-AS1	chr20	62664926	62665280	7.97	exon	2.95121E-10
ENSG00000267056	AC005336.1	chr19	15911291	15911824	7.84	exon	0.010715193
ENSG00000180340	FZD2	chr17	44557755	44558173	-7.77	CDS	1E-12

3'UTR, 3' untranslated region; 5'UTR, 5' untranslated region; CDS, coding sequence; exon, expressed region.

### m6A-Containing Genes are Involved in Important Biological Processes and Pathways

**Figure 2:**
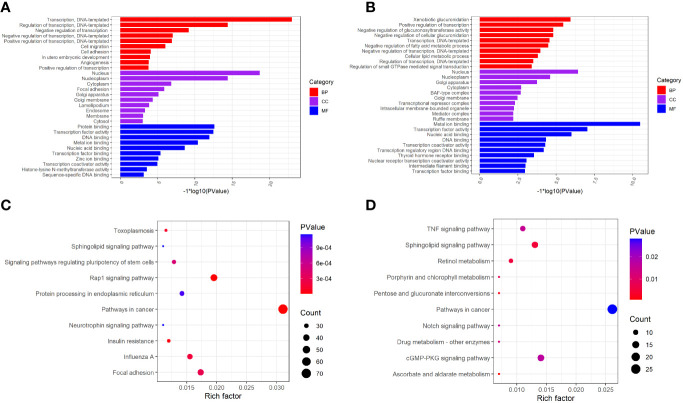
GO function and KEGG pathway enrichment of differently methylated m6A genes. **(A)** The top 10 GO terms of genes with down-regulated m6A peaks. **(B)** The top 10 GO terms of genes with up-regulated m6A peaks. **(C)** The top 10 KEGG pathways of genes with down-regulated m6A peaks. **(D)** The top 10 KEGG pathways of genes with up-regulated m6A peaks.

**Figure 3:**
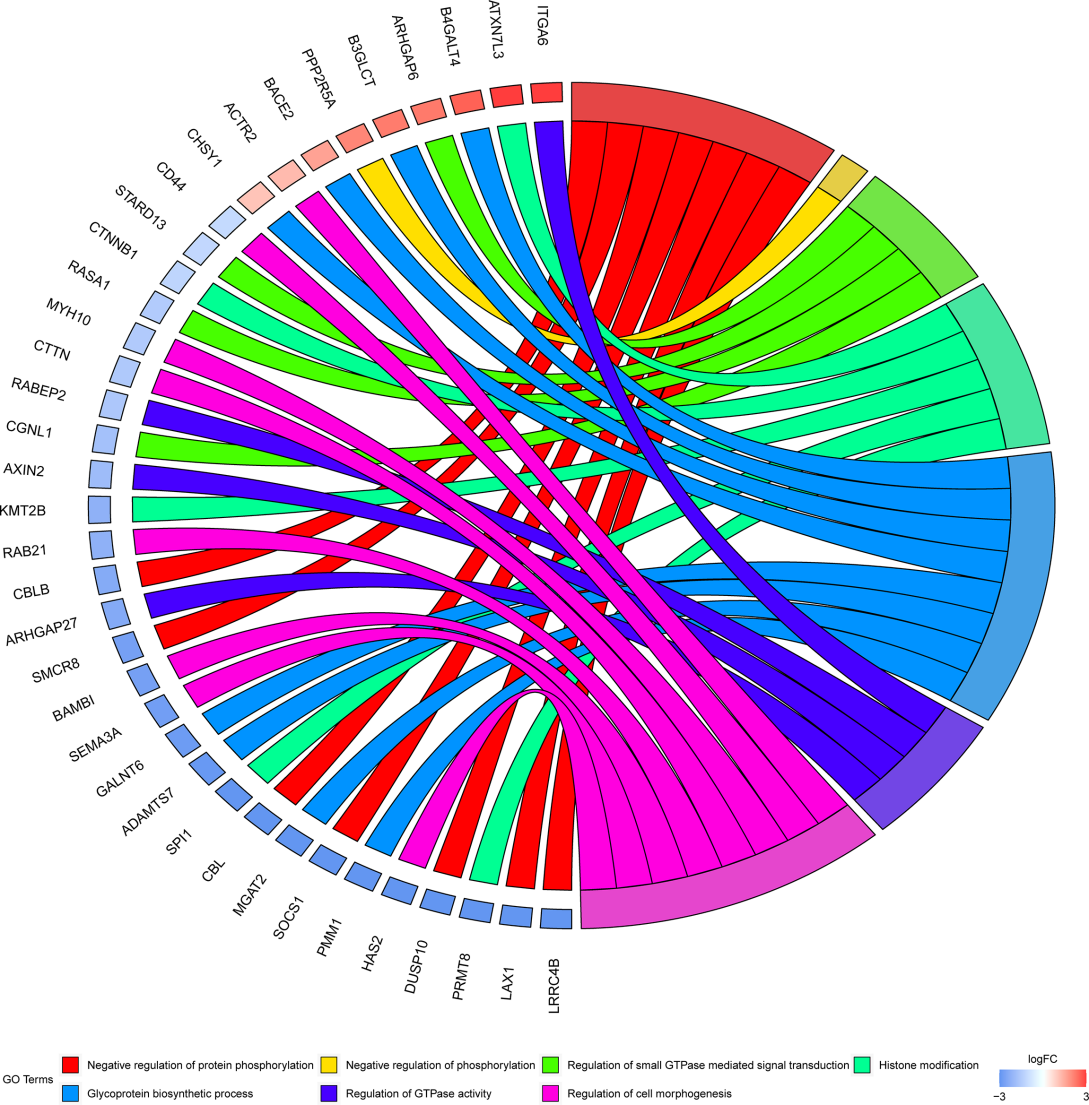
GO terms enrichment analyses of differently methylated genes between in tumor and normal. Squares in the left semicircle refer to the differently methylated mRNAs, and the squares in the right semicircle refer to the GO terms. Fold change ≥ 2 and *P*-value < 0.05, genes compared to GO terms.

To investigate the biological significance of m6A modification in LUAD, GO terms and KEGG pathway analyses of differential methylated genes were performed. For GO analyses, biological processes (BP), cellular components (CC), and molecular functions (MF) were taken into consideration. **Figure 2A** demonstrates the top 10 significantly enriched BP, CC, and MF of genes with downregulated m6A peaks, while GO analysis of genes goes along with upregulated m6A peaks as shown in **Figure 2B**. KEGG pathway analysis showed that genes with downregulated m6A peaks in LUAD were significantly associated with the pathways in cancer, Rap1 signaling pathway, and insulin resistance (**Figure 2C**). Genes with upregulated m6A peaks were significantly associated with the ascorbate and alternate metabolism, pentose and glucuronate interconversions, and sphingolipid signaling pathway (**Figure 2D**). Additionally, in terms of the fold change, differential methylated genes between tumor and normal were associated with the negative regulation of protein phosphorylation, glycoprotein biosynthetic process, and regulation of GTPase activity (**Figure 3**).

### Overview of Transcriptome Profiles and Conjoint Analyses of MeRIP-Seq and RNA-Seq Data

**Figure 4:**
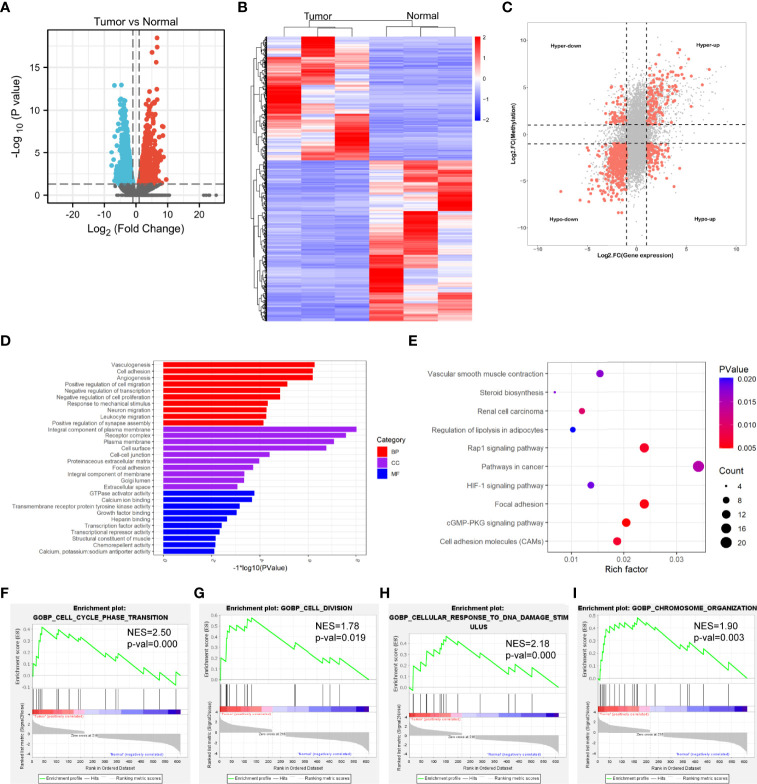
Conjoint analysis of MeRIP-seq and RNA-seq data. **(A)** Volcano plots showing the differentially expressed genes in tumor vs normal. **(B)** Heatmap plots showing the differentially expressed genes in tumor vs normal. **(C)** Four-quadrant plots showing the distribution of genes with significant changes in both the m6A modification and RNA levels. **(D)** The top 10 GO terms of genes with significant changes in both the m6A modification and mRNA levels. **(E)** The top 10 KEGG pathways of genes with significant changes in both the m6A modification and RNA levels. **(F, G, H, I)** GSEA enrichment of genes with significant changes in both the m6A modification and RNA levels.

The transcriptome profiles of tumor tissues versus normal tissues from three LUAD patients were detected using RNA-seq (MeRIP-seq input library). Compared to normal tissues, tumor tissues had 1726 up-regulated genes and 2011 down-regulated genes (**Figures 4A, B**). The top 20 differently expressed genes are listed in **Table 2**. Then, all differential methylated m6A peaks with differential RNA levels (612) were divided into four groups by conjoint analysis for the MeRIP-seq and RNA-seq data. It was found that 177 hypermethylated m6A peaks in RNAs were significantly upregulated (128; hyper-up) or downregulated (49; hyper-down), while 435 hypomethylated m6A peaks in RNAs that were significantly upregulated (88; hypo-up) or downregulated (347; hypo-down) (**Figure 4C**). GO and KEGG pathway analyses for the biological significance of those genes (612) with differential methylated m6A peaks and synchronously differential expression were performed. GO analysis indicated that these genes were mainly enriched in the vasculogenesis (BP), an integral component of the plasma membrane (CC), and GTPase activator activity (MF) (**Figure 4D**). KEGG pathway analysis revealed that these genes were predominately enriched in the cGMP-PKG signaling pathway, focal adhesion, and Rap1 signaling pathway (**Figure 4E**). By contrast, GSEA enrichment of genes with significant changes in both the m6A modification and RNA levels showed that these genes were enriched in cell cycle phase transition, cell division, cellular response to DNA damage stimulus, and chromosome organization (**Figures 4F–I**).

**Table 2:** Top 20 differently genes in m6A-seq&RNA-seq (Tumor/Normal).

Gene ID	Gene name	Chromosome	Peak start	Peak end	Regulation	Peak region	P value
ENSG00000164778	EN2	chr7	155458128	155458456	Hyper-up	5'UTR	2.00E-12
ENSG00000137571	SLCO5A1	chr8	69672400	69673269	Hyper-up	3'UTR	0.007585776
ENSG00000169258	GPRIN1	chr5	176596219	176596548	Hyper-up	3'UTR	5.62E-05
ENSG00000232803	SLCO4A1-AS1	chr20	62664926	62665280	Hyper-up	exon	2.90E-10
ENSG00000086696	HSD17B2	chr16	82098204	82098534	Hyper-up	3'UTR	0.022387211
ENSG00000205334	LINC01460	chr2	27707542	27708020	Hyper-up	exon	0.018197009
ENSG00000155886	SLC24A2	chr9	19515834	19516134	Hyper-up	3'UTR	0.029512092
ENSG00000143297	FCRL5	chr1	157514696	157514967	Hyper-up	3'UTR	0.030902954
ENSG00000259674	AC092868.1	chr15	59407708	59408154	Hyper-up	exon	0.025118864
ENSG00000227640	SOX21-AS1	chr13	94712864	94713313	Hyper-up	exon	0.03801894
ENSG00000147119	CHST7	chrX	46598169	46598408	Hypo-down	3'UTR	0.000616595
ENSG00000155657	TTN	chr2	178535385	178535626	Hypo-down	CDS	1.95E-06
ENSG00000205038	PKHD1L1	chr8	109445040	109445431	Hypo-down	CDS	0.019498446
ENSG00000243910	TUBA4B	chr2	219271798	219272128	Hypo-down	3'UTR	0.021379621
ENSG00000161649	CD300LG	chr17	43862405	43862704	Hypo-down	3'UTR	0.029512092
ENSG00000228401	HSPC324	chr9	136648755	136649020	Hypo-down	exon	0.000977237
ENSG00000177464	GPR4	chr19	45590631	45591111	Hypo-down	CDS	5.25E-06
ENSG00000111834	RSPH4A	chr6	116627847	116628087	Hypo-down	CDS	2.88E-08
ENSG00000206384	COL6A6	chr3	130661905	130662146	Hypo-down	CDS	1.45E-05
ENSG00000185985	SLITRK2	chrX	145822103	145822601	Hypo-down	5'UTR	0.037153523

3'UTR, 3' untranslated region; 5'UTR, 5' untranslated region; CDS, coding sequence; exon, expressed region.

### Identifying the 2 genes Containing m6A Modification Correlates with Clinical Parameters of LUAD Patients

**Figure 5:**
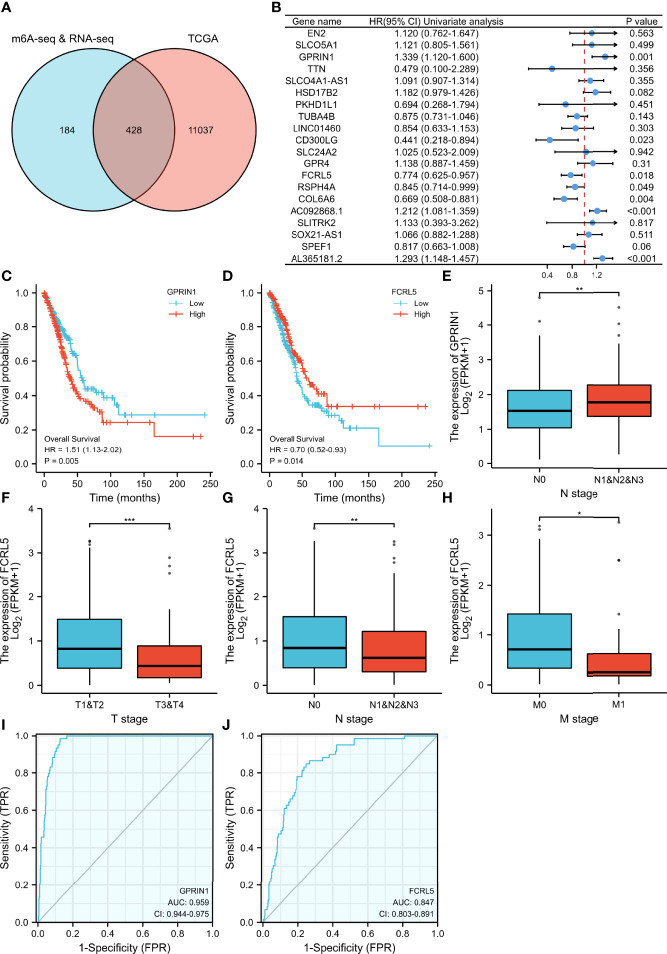
The relationship between gene expression regulated by m6A modification and clinical parameters in the TCGA database. **(A)** The intersection of genes in our study and TCGA database. **(B)** 20 prognostic genes were identified *via* univariate Cox regression analysis. **(C, D)** Overall survival curves of GPRIN1 and FCRL5, respectively. **(E)**The relationships between GPRIN1 expression and N. **(F, G, H)** The relationships between FCRL5 expression and M, N, and clinical stage. **(I, J)** The ROC curves of GPRIN1 and FCRL5, respectively. P*<0.5, P**<0.01, P***<0.001. (N: lymph node metastasis; M: distant metastasis.).

To evaluate the clinical significance of m6A modification regulating gene expression, the TCGA database was explored. 428 differential expressed genes commonly expressed in this study and the TCGA database were identified (**Figure 5A**). The top 20 differently expressed genes are listed in **Table 3**. The univariate Cox regression analysis of the expression of the top 20 differential expressed genes was performed to identify prognostic genes. The results demonstrated that the expression of 7 candidate genes was significantly related to the prognosis (p < 0.05) of LUAD patients (**Figure 5B**). Furthermore, we identified 2 genes (FCRL5 and GPRIN1) correlated with the prognosis of patients with LUAD. The overall survival curves of these 2 genes are shown in **Figures 5C, D**. This result showed that high expression of FCRL5 was associated with a better prognosis for LUAD patients, while high expression of GPRIN1 was associated with a worse prognosis for LUAD patients. The relationship between these 2 genes and clinical features was also analyzed in this study. GPRIN1 was found to be significantly high in patients with lymph node metastasis (**Figure 5E**), while FCRL5 was low expressed in patients with the clinical stage (**Figure 5F**), lymph node metastasis (**Figure 5G**), and distant metastasis (**Figure 5H**). In addition, the relationship between these 2 genes and clinical diagnosis showed that GPRIN1 had high accuracy in predicting the outcome, while FCRL5 had certain accuracy (**Figures 5I, J**). Previously, Li et al. (20) also reported GPRIN1 as a factor for poor prognosis of non-small cell lung cancer by analyzing RNA-seq data in TCGA and GEO databases. This indicates that GPRIN1 is a vital oncogene in LUAD, and m6A modification plays an important role in the expression level of GPRIN1.

**Table 3:** Top 20 differently genes in m6A-seq&RNA-seq&TCGA (Tumor/Normal).

Gene ID	Gene name	Chromosome	Peak start	Peak end	Log2 FC	Peak region	Regulation	P value
ENSG00000164778	EN2	chr7	155458128	155458456	8.97	5'UTR	Hyper-up	1.99526E-12
ENSG00000137571	SLCO5A1	chr8	69672400	69673269	8.62	3'UTR	Hyper-up	0.007585776
ENSG00000169258	GPRIN1	chr5	176596219	176596548	8.43	3'UTR	Hyper-up	5.62341E-05
ENSG00000155657	TTN	chr2	178535385	178535626	-8.39	CDS	Hypo-down	1.94984E-06
ENSG00000232803	SLCO4A1-AS1	chr20	62664926	62665280	7.97	exon	Hyper-up	2.95121E-10
ENSG00000086696	HSD17B2	chr16	82098204	82098534	7.44	3'UTR	Hyper-up	0.022387211
ENSG00000205038	PKHD1L1	chr8	109445040	109445431	-7.23	CDS	Hypo-down	0.019498446
ENSG00000243910	TUBA4B	chr2	219271798	219272128	-7.11	3'UTR	Hypo-down	0.021379621
ENSG00000205334	LINC01460	chr2	27707542	27708020	7.09	exon	Hyper-up	0.018197009
ENSG00000161649	CD300LG	chr17	43862405	43862704	-7.06	3'UTR	Hypo-down	0.029512092
ENSG00000155886	SLC24A2	chr9	19515834	19516134	7.03	3'UTR	Hyper-up	0.029512092
ENSG00000177464	GPR4	chr19	45590631	45591111	-7.03	CDS	Hypo-down	5.24807E-06
ENSG00000143297	FCRL5	chr1	157514696	157514967	6.98	3'UTR	Hyper-up	0.030902954
ENSG00000111834	RSPH4A	chr6	116627847	116628087	-6.97	CDS	Hypo-down	2.88403E-08
ENSG00000206384	COL6A6	chr3	130661905	130662146	-6.95	CDS	Hypo-down	1.44544E-05
ENSG00000259674	AC092868.1	chr15	59407708	59408154	6.87	exon	Hyper-up	0.025118864
ENSG00000185985	SLITRK2	chrX	145822103	145822601	-6.69	5'UTR	Hypo-down	0.037153523
ENSG00000227640	SOX21-AS1	chr13	94712864	94713313	6.66	exon	Hyper-up	0.03801894
ENSG00000101222	SPEF1	chr20	3777799	3778552	-6.63	exon	Hypo-down	1.99526E-11
ENSG00000272068	AL365181.2	chr1	156637991	156638409	6.62	exon	Hyper-up	0.029512092

3'UTR, 3' untranslated region; 5'UTR, 5’ untranslated region; CDS, coding sequence; exon, expressed region.

**Figure 6:**
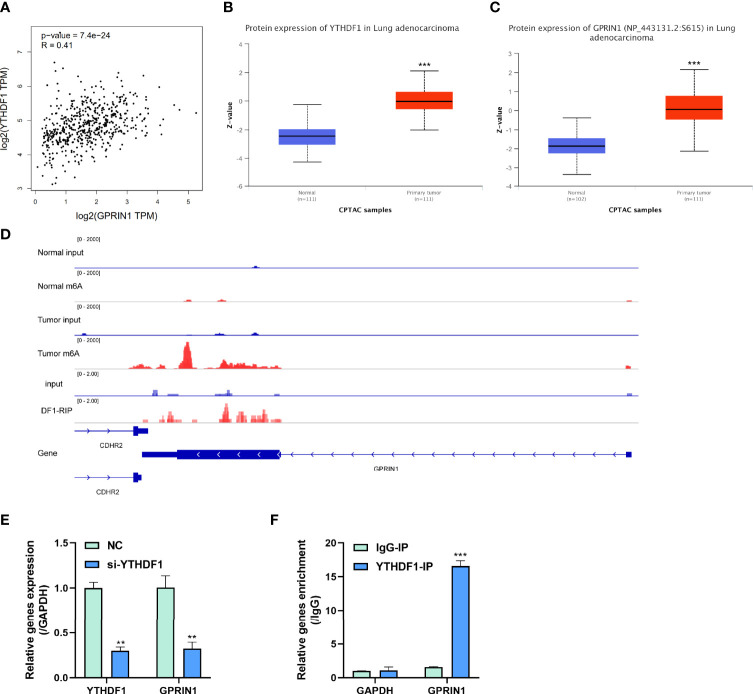
m6A reader YTHDF1 regulated GPRIN1 expression. **(A)** A strong positive correlation between YTHDF1 and GPRIN1 in the GEPIA2 database. **(B, C)** The protein expression of YTHDF1 and GPRIN1 in the CPTAC database, respectively. **(D)** Tumor m6A signal was increased at specific positions 3’UTR of GPRIN1. The region with a gain of m6A in LUAD is the same that gains enrichment of YTHDF1 in A549 cells. **(E)** Knockdown of YTHDF1 downregulated the RNA levels of GPRIN1 in LUAD A549 cells. **(F)** RIP-derived RNA in A549 cells was measured by RT-qPCR. P*<0.5, P**<0.01, P***<0.001.

To explore the potential roles of m6A modification in GPRIN1 expression, we conducted a bioinformatics analysis using the GEPIA2 and CPTAC databases. It was found that GPRIN1 was strongly correlated with m6A reader YTHDF1 (*r* = 0.41) (**Figure 6A**). Also, both GPRIN1 and YTHDF1 proteins were highly expressed in LUAD (**Figures 6B, C**). Further, the m6A RIP-seq data showed that the m6A peaks were enriched in the 3’UTRs of GPRIN1 transcript in LUAD tissues. Compared to the normal tissues, m6A methylation was up-regulated in the vicinity of the 3’UTR of GPRIN1. The region with a gain of m6A in LUAD is the same that gains enrichment of YTHDF1 in A549 cells (**Figure 6D**). It was reported that YTHDF1 regulated gene expression *via* control of mRNA translation efficiency in an m6A-dependent manner (21). To evaluate whether YTHDF1 involved the expression of GPRIN1, siRNAs targeting YTHDF1 were transfected efficiently into A549 cells. As shown in **Figure 6E**, knockdown YTHDF1 significantly down-regulated the expression level of GPRIN1. Furthermore, we verified that YTHDF1 could bind GPRIN1 mRNA by DF1-RIP-qPCR assay, in which the fold enrichment of the YTHDF1-IP group was significantly higher than that of the IgG-IP group (**Figure 6F**). Therefore, we hypothesized that YTHDF1 regulates the expression of GPRIN1 by recognizing the m6A modification on GPRIN1 mRNA.

## Discussion

m6A is the most abundant internal modification on eukaryotic mRNAs. Recently, the significance of m6A modification is revealed that it involves almost all aspects of mRNA metabolism, including pre-mRNA processing, splicing, nuclear export, translation, and degradation (22). m6A modification also plays critical roles in physiology and pathology such as embryonic development (23), neurodevelopment (24), immunoregulation (25), and tumorigenesis (26). It is also reported that m6A modification plays a crucial role in the development and progression of LUAD (16, 27). However, the transcriptome-wide m6A methylome of LUAD has not been characterized. In this study, MeRIP-seq and RNA-seq were performed to reveal the m6A mapping in LUAD. We identified 4041 anomalously regulated m6A peaks in tumor tissues, of which 1192 m6A peaks were upregulated and 2849 m6A peaks were downregulated. Moreover, GO and KEGG pathway analyses revealed the potential functions of differential methylated transcripts. Additionally, by combining MeRIP-seq and RNA-seq data, genes with differential methylated m6A peaks and synchronously differential expression were enriched in cancer-related pathways. Finally, 2 genes were further proven to be associated with the prognosis and diagnosis of LUAD patients in the TCGA database.

In this study, it was detected that differential methylated RNAs related to important biological pathways *via* KEGG pathway analysis. It was found that upregulated m6A modification genes were associated with the ascorbate and alternate metabolism, pentose, and glucuronate interconversions, and sphingolipid signaling pathway. Moreover, downregulated m6A modification genes were significantly associated with the pathways in cancer, Rap1 signaling pathway, and insulin resistance.

Combining MeRIP-seq and RNA-seq data, 612 genes with differential methylated m6A peaks and synchronously differential expressed in RNA were identified. We performed GO and KEGG pathway analyses to investigate the biological significance of those genes. KEGG pathway analysis revealed that these genes were primarily enriched in the cGMP-PKG signaling pathway, focal adhesion, and Rap1 signaling pathway. It was revealed that these biological pathways are closely implicated in the onset and development of tumors, including tumor growth, and metastasis (28, 29). Rap1 is activated in response to upstream signalings, such as growth factors, cytokines, and chemokines that act on receptor tyrosine kinases and G-protein coupled receptors (30). Rap1 plays an important role in cell-matrix adhesion and supports the key role of the RAP1/RAC1 signaling axis in head and neck squamous cell carcinoma (HNSCC) cell migration *via* inducing α5β1 integrin *via* the extracellular matrix molecule fibronectin (31). Rap1 activity is increased in progressively metastatic prostate cancer cell lines and promotes metastasis in *in vivo* models through the integrins (32). Xiang et al. found that H. pylori infection enhances PRTG expression by promoting the stabilization of transcription factor ZEB1 and recruitment of PRTG promoters, and subsequently activates the cGMP/PKG signaling pathway to further promote proliferation, metastasis, and chemotherapy resistance of gastric cancer cells (33). The effects of EMX2OS and FUS on proliferation, invasion, and migration of human cell lines DU145 and PC3 mediated by the cGMP-PKG pathway were investigated by functional gain and functional loss experiments (34). The attenuation of cellular β-catenin accumulation through the activated cGMP-PKG pathway and the increased phosphorylation of β-catenin were observed in exisulind-treated colonic tumor cells (35).

In further investigation, 2 genes (FCRL5 and GPRIN1) were identified as most correlated with the development of LUAD using TCGA databases. Overexpression of GPRIN1 served as a factor for poor prognosis, while FCRL5 favors prognosis. Besides, high expression of GPRIN1 is highly involved in the lymph node metastasis, and FCRL5 with not only lymph node metastasis but also distant metastasis and the clinical stage of LUAD patients. Further analysis revealed that the region with a gain of m6A in LUAD is the same that gains enrichment of YTHDF1 in A549 cells. What’s more, YTHDF1 binds GPRIN1 mRNA by YTHDF1-RIP-qPCR assay, in which the fold enrichment of the YTHDF1-IP group was significantly higher than that of the IgG-IP group.

However, our research still has some shortcomings. Firstly, LUAD is a kind of highly heterogeneous cancer, and the tissue samples of clinical patients vary greatly. Although the three pairs of clinical samples met the statistical requirements, they were too small a sample size to reflect the differential m6A modification and gene expression between LUAD and adjacent tissue in the LUAD population, and the number of cases still must be further increased. Secondly, although we have performed some experiments to verify that YTHDF1 regulates GPRIN1 expression, more experiments are needed to strengthen the credibility, which is also the direction of our future in-depth research.

In summary, the different m6A methylome in LUAD relative to the corresponding normal controls was analyzed, which demonstrated a strong association between m6A modifcation and LUAD progression. 2 genes (FCRL5 and GPRIN1) with differential methylated m6A peaks and synchronously differential expression were associated with the prognosis and diagnosis of LUAD patients. Further one step research, GPRIN1 was found to act as an downstream target of YTHDF1. Targeting the YTHDF1-m6A-GPRIN1 axes may offer a promising therapeutic approach in curing LUAD.

## Data Availability Statement

The data presented in the study are deposited in the GEO repository, accession number GSE198288.

## Ethics Statement

The studies involving human participants were reviewed and approved by the Ethics Committee of the South University of Science and Technology Hospital. The patients/participants provided their written informed consent to participate in this study.

## Author Contributions

WM was a major contributor to all of the experimental work, data analysis, and manuscript writing. KW, YZ, and WL were involved in the experimental work. QY, QM, NW, and GZ were involved in the data analysis. YW acquired funding and assisted with the manuscript development. The final manuscript was reviewed and approved by all of the authors.

## Funding

This work was supported by the Stability Support Plan for Higher Education Institutions in Shenzhen (20200809124551001).

## Conflict of Interest

The authors declare that the research was conducted in the absence of any commercial or financial relationships that could be construed as a potential conflict of interest.

## Publisher’s Note

All claims expressed in this article are solely those of the authors and do not necessarily represent those of their affiliated organizations, or those of the publisher, the editors and the reviewers. Any product that may be evaluated in this article, or claim that may be made by its manufacturer, is not guaranteed or endorsed by the publisher.
