# Corrigendum: Comprehensive analysis of the transcriptome-wide m6A methylome in lung adenocarcinoma by MeRIP sequencing

**DOI:** 10.3389/fonc.2022.1032295

**Published:** 2022-10-04

**Authors:** Wenli Mao, Qingzhen Yu, Kefeng Wang, Qiang Ma, Yuxin Zheng, Guojun Zhang, Wei Luo, Nianwu Wang, Yukun Wang

**Affiliations:** ^1^ Department of Pharmacology, School of Medicine, Southern University of Science and Technology, Shenzhen, China; ^2^ Medical Research Center, Southern University of Science and Technology Hospital, Shenzhen, China; ^3^ Nutrition Department, Southern University of Science and Technology Hospital, Shenzhen, China; ^4^ Department of Clinical Laboratory, Southern University of Science and Technology Hospital, Shenzhen, China; ^5^ Department of Pharmacy, Southern University of Science and Technology Hospital, Shenzhen, China

**Keywords:** lung adenocarcinoma, m6A, MeRIP-seq, GPRIN1, prognosis

In the published article, there was an error in **Figure 2B** as published. The corrected **Figure 2B** and its caption “GO function and KEGG pathway enrichment of differently methylated m6A genes. **(A)** The top 10 GO terms of genes with down-regulated m6A peaks. **(B)** The top 10 GO terms of genes with up-regulated m6A peaks. **(C)** The top 10 KEGG pathways of genes with down-regulated m6A peaks. **(D)** The top 10 KEGG pathways of genes with up-regulated m6A peaks.” appear below.

**Figure 2:**
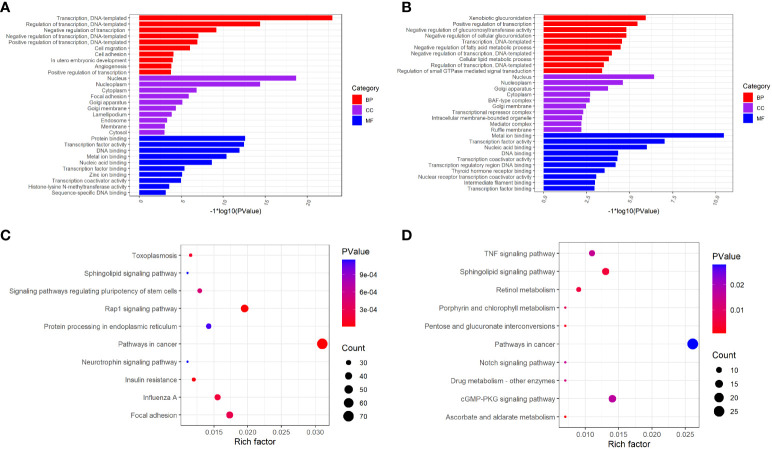
GO function and KEGG pathway enrichment of differently methylated m6A genes. **(A)** The top 10 GO terms of genes with down-regulated m6A peaks. **(B)** The top 10 GO terms of genes with up-regulated m6A peaks. **(C)** The top 10 KEGG pathways of genes with down-regulated m6A peaks. **(D)** The top 10 KEGG pathways of genes with up-regulated m6A peaks.

The authors apologize for this error and state that this does not change the scientific conclusions of the article in any way. The original article has been updated.

## Publisher’s note

All claims expressed in this article are solely those of the authors and do not necessarily represent those of their affiliated organizations, or those of the publisher, the editors and the reviewers. Any product that may be evaluated in this article, or claim that may be made by its manufacturer, is not guaranteed or endorsed by the publisher.

