# Supplementary figures and images for: Comprehensive Analysis of the Transcriptome-wide m6A Methylome in Lung Adenocarcinoma by MeRIP Sequencing
Source: Front Oncol. 2022 Jul 11;12:791332. doi: 10.3389/fonc.2022.791332 (PMC9315447; doi:10.3389/fonc.2022.791332)

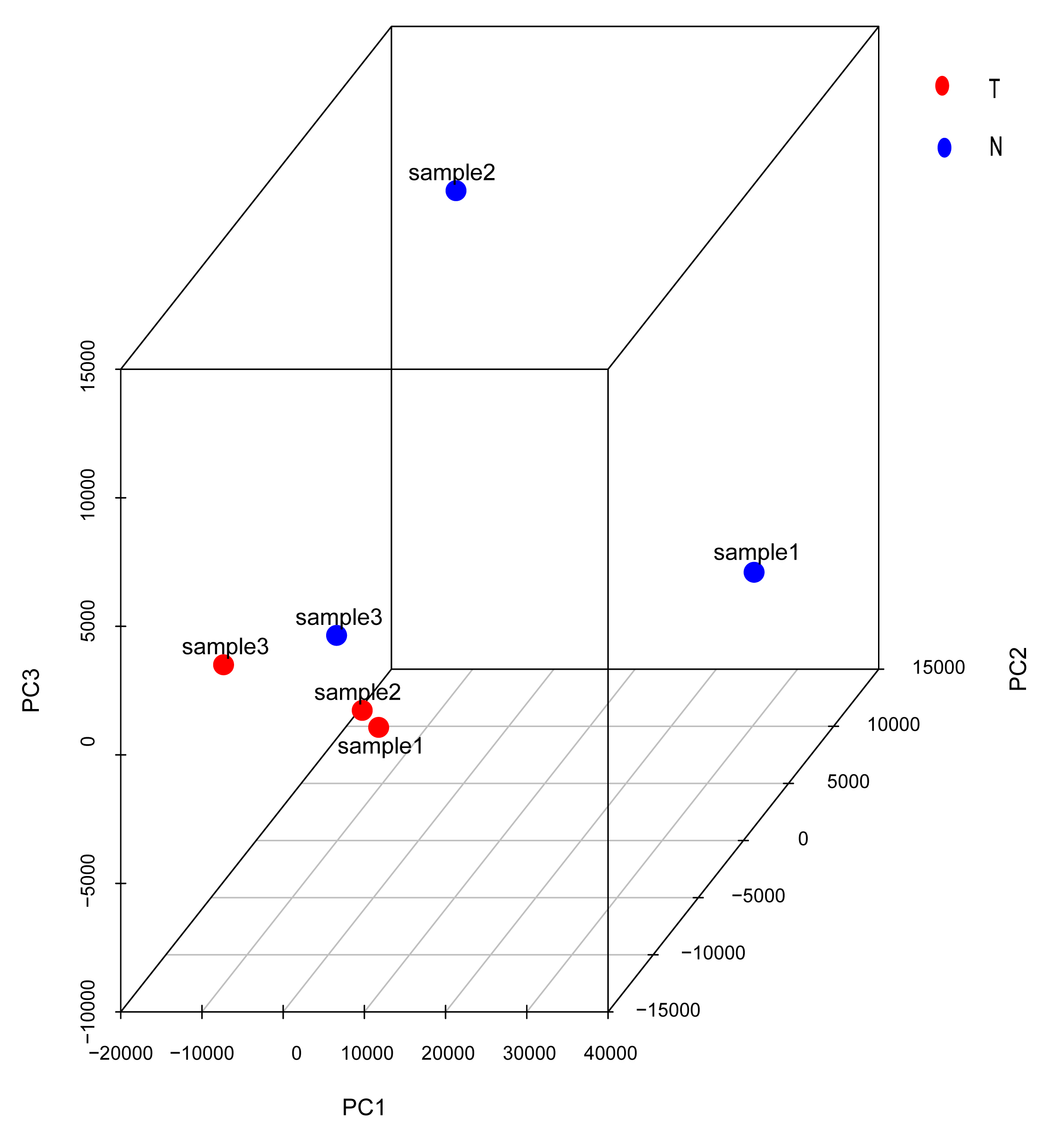

Supplement: Supplementary file 1 [file Image_1.tif]
